# Sparse matrix computations for dynamic network centrality

**DOI:** 10.1007/s41109-017-0038-z

**Published:** 2017-06-24

**Authors:** Francesca Arrigo, Desmond J. Higham

**Affiliations:** University of Strathclyde, 16 Richmond St, Glasgow, G1 1XQ UK

**Keywords:** Dynamic network, Sparsification, Centrality, Katz centrality, Social network analysis

## Abstract

Time sliced networks describing human-human digital interactions are typically large and sparse. This is the case, for example, with pairwise connectivity describing social media, voice call or physical proximity, when measured over seconds, minutes or hours. However, if we wish to quantify and compare the overall time-dependent centrality of the network nodes, then we should account for the global flow of information through time. Because the time-dependent edge structure typically allows information to diffuse widely around the network, a natural summary of sparse but dynamic pairwise interactions will generally take the form of a large dense matrix. For this reason, computing nodal centralities for a time-dependent network can be extremely expensive in terms of both computation and storage; much more so than for a single, static network. In this work, we focus on the case of dynamic communicability, which leads to broadcast and receive centrality measures. We derive a new algorithm for computing time-dependent centrality that works with a sparsified version of the dynamic communicability matrix. In this way, the computation and storage requirements are reduced to those of a sparse, static network at each time point. The new algorithm is justified from first principles and then tested on a large scale data set. We find that even with very stringent sparsity requirements (retaining no more than ten times the number of nonzeros in the individual time slices), the algorithm accurately reproduces the list of highly central nodes given by the underlying full system. This allows us to capture centrality over time with a minimal level of storage and with a cost that scales only linearly with the number of time points. We also describe and test three variants of the proposed algorithm that require fewer parameters and achieve a further reduction in the computational cost.

## Introduction

In network science, centrality measures assign to each node a value that summarizes some aspect of its relative importance. Such measures arose in the social sciences, but have now become very widely used by researchers who wish to summarize important features of large, complex networks (Estrada 2010; Newman 2010; Wasserman and Faust 1994). Because matrix representations of networks are typically sparse, and because centrality measures typically involve the solution of linear systems or eigenvalue problems, it is feasible to compute centrality measures on a current desktop computer for networks with, say, a number of nodes in the millions.

Our focus in this work is the case of time-dependent network sequences (Holme and Saramäki 2011). Such data sets may be regarded as three-dimensional tensors, where, along with the (*i*,*j*) coordinates that capture pairwise connectivity, we also have a third coordinate that represents time (Acar et al. 2009). These types of connections arise, for example, when we record human-human digital interaction through social media, telecommunication or physical proximity. In (Grindrod et al. 2011) the concept of a *dynamic communicability matrix* was introduced, which converted the time sequence of networks into a single two-dimensional array, with (*i*,*j*) element summarizing the ability of node *i* to communicate with node *j*, using the time-dependent sequence of edges recorded in the data. From this matrix, it is straightforward to compute centrality measures:

- *dynamic broadcast centrality* takes large values for nodes that are effective at distributing information,
- *dynamic receive centrality* takes large values for nodes that are effective at gathering information.

In a case study on Twitter data, this approach was seen to be successful, in the sense of correlating well with the independent views of social media experts (Lafin et al. 2013). It was also found to outperform the crude alternative of simply aggregating all edges into a single static network that forgets the time-ordering of the interactions; see (Mantzaris and Higham 2016) for further discussion. Tests in (Chen et al. 2016; Mantaris and Higham 2013) also showed that dynamic broadcast centrality can be effective at quantifying the potential for the spread of disease across time-ordered interactions. In (Fenu and Higham 2017) it is shown how to perform dynamic communicability computations via a large block matrix that is amenable to modern iterative techniques. A weaker version of dynamic broadcast communicability, essentially given by applying the sign function, was proposed in (Lentz et al. 2013) to quantify what those authors term accessibilty.

As we explain in the next section, the computation of dynamic centrality can be expensive in terms of both storage and computational effort, as a result of inevitable matrix fill-in as temporal information accumulates. Our overall aim here is to address this issue by deriving a new algorithm that delivers good approximations to the original dynamic broadcast centrality measure while retaining the benefits of the sparsity present in the time slices.

We note that other approaches to computation of node centrality for time-dependent networks have been put forward. For example, (Tang et al. 2009; 2010a, b) made use of paths rather than walks, which, for our purposes, leads to an infeasibly expensive algorithm. In (Taylor et al. 2017) a block-matrix approach was suggested which allows centrality measures for static networks to be applied. However, as mentioned in (Mantzaris and Higham 2016), that formulation does not fully respect the arrow of time.

Compared with the earlier conference paper (Arrigo and Higham 2017), this article includes further computational results and accompaining discussions, and in particular has a new section (“Further reduction” section) that shows how performance can be improved by reducing the number of parameters.

## Background and notation

In this section we recall some definitions and notation that will be used throughout. Let *t*_0_<*t*_1_<⋯<*t*_*M*_ be an ordered sequence of time points and let $\left \{{\mathcal {G}}^{[k]}\right \}_{k=0}^{M} = \left \{\left ({\mathcal {V}}^{[k]},{\mathcal {E}}^{[k]}\right)\right \}$ be a time-ordered sequence of unweighted graphs defined over *n* nodes. A graph is said to be unweighted when all its edges have the same weight, which can thus be assumed to be unitary. Consider the adjacency matrices $\left \{A^{[k]}\right \}_{k=0}^{M} = \left \{ \left (a^{[k]}_{ij}\right)\right \}\in {\mathbb {R}^{n\times n}}$ associated with these graphs at times $\{ t_{k} \}_{k=0}^{M}$, whose entries are defined as

$$a^{[k]}_{ij} = \left\{ \begin{array}{ll} 1 & \text{if } (i,j)\in{\mathcal{E}}^{[k]} \\ 0 & \text{otherwise.} \end{array}\right. $$

In (Grindrod et al. 2011) the concept of a *dynamic walk of length p* was introduced to extend to the temporal case the well-known concept of a walk of length *p* in static networks. Loosely, we have a (possibly repeated) sequence of *p*+1 nodes connected by edges that appear in a suitable order. More precisely, a dynamic walk of length *p* from node *i*_1_ to node *i*_*p*+1_ consists of a sequence of nodes *i*_1_,*i*_2_,…,*i*_*p*+1_ and a sequence of times $t_{r_{1}}\leq t_{r_{2}}\leq \cdots \leq t_{r_{p}}$ such that $a^{[r_{m}]}_{i_{m}i_{m+1}} \neq 0$ for *m*=1,2,…,*p*. We stress that more than one edge can share a time slot, and that time slots must be ordered but do not need to be consecutive.

The concept of dynamic walk was used to motivate the definition of the *dynamic communicability matrix*

1a $$ Q^{[M]} = \left(I-\alpha A^{[0]}\right)^{-1}\left(I-\alpha A^{[2]}\right)^{-1}\cdots\left(I-\alpha A^{[M]}\right)^{-1},  $$

which can be defined equivalently via the iteration

1b $$ Q^{[k]} = Q^{[k-1]}\left(I-\alpha A^{[k]}\right)^{-1}, \quad k=0,1,\ldots,M,  $$

where *Q* ^[−1]^=*I* is the identity matrix of order *n*, 0<*α*<1/*ρ* ^∗^, and $\rho ^{*}=\max \limits _{k=0:M}\left \{\rho \left (A^{[k]}\right)\right \}$ is the largest spectral radius among the spectral radii of the matrices {*A* ^[*k*]^}. Here the free parameter *α* plays the same role as in the classical Katz centrality measure for static networks (Estrada 2010; Katz 1953; Newman 2010). For simplicity, our notation does not explicitly record the dependence of *Q* upon *α*.

To avoid overflow in the computations, a normalization step *Q*↦*Q*/∥*Q*∥ should follow each iteration in (1b). Throughout this work we use the Euclidean norm.

The requirement *α*<1/*ρ* ^∗^ ensures that the resolvents in (1a) exist and can be expanded as $\left (I-\alpha A^{[k]}\right)^{-1} = \sum _{p=0}^{\infty } \left (\alpha A^{[k]}\right)^{p}$. It follows that the entries of *Q* ^[*k*]^ provide a weighted count of the dynamic walks between any two nodes in the networks using the ordered sequence of matrices *A* ^[0]^,*A* ^[1]^,…,*A* ^[*k*]^, weighting walks of length *p* by a factor *α* ^*p*^. Hence, (*Q* ^[*k*]^)_*ij*_ is an overall measure of the ability of node *i* to send messages to node *j*.

Using the dynamic communicability matrix one can define and compare the broadcast and receive centrality of nodes by taking row and column sums of the matrix *Q* ^[*M*]^, respectively. The *broadcast centrality* of node *i* is defined as $b_{i}^{[M]}:=\textbf {e}_{i}^{T} Q^{[M]}\mathbf {1}$, where $\textbf {e}_{i}\in \mathbb {R}^{n}$ is the *i*th column of *I*, the superscript “*T*” denotes transposition, and $\mathbf {1}\in \mathbb {R}^{n}$ is the vector of all ones. Similarly, the *receive centrality* of node *j* is defined as $r_{j}^{[M]}:=\mathbf {1}^{T} Q^{[M]}\textbf {e}_{j}$. It is straightforward to show that the latter satisfies a lower-dimensional, vector-valued iteration given by

$$\textbf{r}^{[k]}:= \mathbf{1}^{T} Q^{[k]} = \textbf{r}^{[k-1]}\left(I-\alpha A^{[k]}\right)^{-1}, \quad k= 0,1,\ldots M. $$

The receive centrality of the nodes can thus be updated at each step by solving a single sparse linear system whose coefficient matrix is the latest network time slice. In particular, this means that we do not need to store and update the full matrix *Q* ^[*k*]^ to recover the receive centrality of nodes at level *k*. By contrast, to compute the broadcast centrality vector, **b** ^[*M*]^=*Q* ^[*M*]^ **1**, we need access to the current dynamic communicability matrix at each step. Intuitively, this difference arises because,

- given a summary of how much information is flowing *into* each node, we can propagate this information forward when new edges emerge: receive centrality cares about where the information *terminates*, but
- a summary of how much information is flowing *out of* each node cannot be straightforwardly updated when new edges emerge: broadcast centrality cares about where the information *originates*.

Our focus here is on the natural setting where data is processed sequentially, with the centrality scores being updated as each new time slice *A* ^[*k*]^ arrives. As confirmed in “Numerical tests” section on some real data sets, we then face a fundamental issue with the use of the dynamic communicability matrix: although the time slices are typically sparse, *Q* ^[*k*]^ generally evolves into a dense matrix. At this stage, computing dynamic communicability from (1b) requires us to store a full *O*(*n* ^2^) matrix and solve at each subsequent time point a corresponding full linear system. In the next section, we therefore develop and justify an approximation where matrix fill-in is controlled so that the benefits of sparse matrix storage and computation are recovered.

## Sparsification

To create a sparse approximation, $\widehat {Q}^{[k]}$, to the dynamic communicability matrix, *Q* ^[*k*]^, we first observe that the original iteration (1b) includes some traversals that are not very meaningful, e.g., repeated cycles *i*→*j*→*i*→*j*→*i*→*j* using the same undirected edge at the same time point. We thus use an “at most one edge per time point” alternative to (1b) so as to avoid considering these types of walks and similar ones:

2 $$ \widehat{Q}^{[k]} = \widehat{Q}^{[k-1]}\left(I+\alpha A^{[k]}\right), \quad k=0,1,\ldots,M,  $$

with $\widehat {Q}^{[-1]} = I$ and *α*<1/*ρ* ^∗^, as before. As discussed in (Grindrod et al. 2011), this matrix product can be interpreted in terms of network combinatorics; at each time step a dynamic traversal can either wait, as described by the identity matrix *I*, or take a current edge, as described by latest adjacency matrix, *A* ^[*k*]^. In the latter case, the length of the walk (i.e., the number of edges used) has increased by one, and thus we multiply the corresponding matrix by *α*. An alternative interpretation is that we are using a second order Taylor approximation for each of the resolvents appearing in (1a). This simplification is likely to be reasonable when either (a) *α* is chosen to be small, so that short walks are favoured, or (b) the powers of *A* ^[*k*]^ do not grow rapidly with *k* (which is typically the case for sparse matrices).

As the time index *k* increases in (2) the number of nonzeros cannot decrease, and the matrix $ \widehat {Q}^{[k]} $ will generally fill in. In order to produce a sparse approximation we will proceed iteratively. At each step we threshold the matrix at a level *θ*_*k*_—this type of approach has been widely used in large scale machine learning, data mining, and signal processing; see, e.g., (Achlioptas et al. 2013; Arora et al. 2006) and references therein. Hence, for *k*=0,1,…,*M* we redefine the iteration to be

3 $$ \widehat{Q}^{[k]} =\frac{\left\lfloor\widehat{Q}^{[k-1]}\left(I + \alpha A^{[k]}\right)\right\rfloor_{\theta_{k}}} {\left\|\left\lfloor\widehat{Q}^{[k-1]}\left(I + \alpha A^{[k]}\right)\right\rfloor_{\theta_{k}}\right\|_{2}},   $$

where $\widehat {Q}^{[-1]} = I$ and for any nonnegative matrix *C*, the matrix $\left \lfloor C \right \rfloor _{\theta _{k}}$ arises from setting to zero all entries where *c*_*ij*_≤*θ*_*k*_.

### **Remark 1**

The matrices $\left \{\widehat {Q}^{[k]}\right \}_{k=0}^{M}$ are nonnegative by construction.

### A little twist

From a network science perspective, the approach just presented has a strong limitation. Imagine a user *i* of Twitter who remains inactive for a long time after each tweet. After such inactivity, the thresholding may zero out all entries in the *i*th row of one of the matrices $\widehat {Q}^{[k]}$. From that time, the *i*th row of the matrices appearing in (3) will always be zero, and no subsequent activity of node *i* will be registered by this approach.

To mitigate pathological behaviour of this type, we modify (3) so as to keep track at each step of the behaviour of those nodes corresponding to zero rows in the iteration matrix. Our final version of the iteration goes as follows:

4 $$ \widehat{Q}^{[k]} = \left\lfloor \widehat{Q}^{[k-1]}\left(I+\alpha A^{[k]}\right)\right\rfloor_{\theta_{k}} + m_{k}{\mathcal{A}}^{[k]}, \quad k=0,1,\ldots,M,  $$

followed by normalization, where $\widehat {Q}^{[-1]}= I$, *m*_*k*_ is the smallest nonzero entry of $\left \lfloor \widehat {Q}^{[k-1]}\left (I+\alpha A^{[k]}\right)\right \rfloor _{\theta _{k}}$, ${\mathcal {A}}^{[k]} = \alpha W^{[k]}A^{[k]}$, and $W^{[k]} = \text {diag}\left (w_{1},w_{2},\ldots,w_{n}\right)\in {\mathbb {R}^{n\times n}}$ is a diagonal matrix whose entries are

$$w_{i} = \left\{ \begin{array}{ll} 1 & \text{if } \textbf{e}_{i}^{T}\left\lfloor \widehat{Q}^{[k-1]}\left(I+\alpha A^{[k]}\right)\right\rfloor_{\theta_{k}}\mathbf{1} = 0 \\ 0 & \text{otherwise.} \end{array} \right. $$

The matrix ${\mathcal {A}}^{[k]}$ keeps track of those edges that appear at step *k* and would otherwise get lost. Indeed, the matrix product *W* ^[*k*]^ *A* ^[*k*]^ returns a matrix that has nonzero entries (if any) only in the rows corresponding to those nodes that have either been inactive until step *k* or have broadcast very little information (which thus was thresholded in a previous iteration). The penalisation by *α* is added because we are taking one hop in the network. Finally, the multiplication by *m*_*k*_ comes from the fact that a poor choice of the parameter *α* may compromise the results. Indeed, the entries of ${\mathcal {A}}^{[k]}$ may be too large with respect to those appearing in $\left \lfloor \widehat {Q}^{[k-1]}\left (I+\alpha A^{[k]}\right)\right \rfloor _{\theta _{k}}$, thus leading to a complete reshaping of the rankings. We refer the reader to “Numerical tests” section for an example of this issue.

#### **Remark 2**

It is possible for the contribution added by $m_{k}{\mathcal {A}}^{[k]}$ to be zero. This happens when the zero rows in $\left \lfloor \widehat {Q}^{[k-1]}\left (I+\alpha A^{[k]}\right)\right \rfloor _{\theta _{k}}$ correspond to nodes that are not broadcasting information at step *k*.

#### **Remark 3**

Note that if *A* ^[*k*]^=0 for some *k*, then $\widehat {Q}^{[k]} = \widehat {Q}^{[k-1]}$, just as *Q* ^[*k*]^=*Q* ^[*k*−1]^.

### On the thresholding parameters

The thresholding parameters {*θ*_*k*_} are a key part of the sparsification process. Before explaining how we select these values in applications, we first describe the types of contributions that are removed from the approximation to the dynamic communicability matrix when the thresholding is performed. There are two key circumstances where the thresholding has an effect:

- the value of *α* ^*p*^ dominates the contribution given by the products of the adjacency matrices, i.e., there are not too many walks of length *p* between the two nodes under consideration;
- the information has not moved from a certain node for a long time and the normalization step has made the corresponding contribution smaller than the other entries.

In both cases, we are dismissing information that has little potential, as it is not diffused much. Clearly, an over-stringent selection of the parameters *θ*_*k*_ may lead to an excessive penalization of these two types of behaviours. Our strategy is to make an initial choice for the maximum number of nonzeros that we will allow in the matrices $\widehat {Q}^{[k]}$, for *k*=0,1,…,*M*. Then, as the iteration proceeds, the thresholding value *θ*_*k*_ is chosen so as to make $\left \lfloor \widehat {Q}^{[k-1]}\left (I + \alpha A^{[k]}\right)\right \rfloor _{\theta _{k}}$ have approximately this desired level of sparsity.

We point out that the maximum number of nonzeros one wants to allow has to be at least *n*+nnz(*A* ^[0]^), where nnz(*A* ^[0]^) is the number of nonzeros in the matrix *A* ^[0]^. Consequently, *θ*_0_<*α*. Indeed, if this is not the case, then we will have *θ*_*k*_≥*α* for all *k* and therefore that $\widehat {Q}^{[k]} = I$ for all *k*.

### Cost comparison

We are now in a position to quantify, at least approximately, the computational benefits of using $\widehat {Q}^{[k]}$ in (4) rather than the exact matrix *Q* ^[*k*]^ in (1b) to compute dynamic broadcast communicability. We will assume that at any time point all nodes have bounded out-degree (independently of the number of nodes, *n*), so that there is a bounded number of nonzeros per row in each *A* ^[*k*]^. Our choice of thresholding parameters will then force $\widehat {Q}^{[k]}$ to have the same property. Because the exact representation *Q* ^[*k*]^ becomes full in general, it follows that:

- We have reduced storage requirements by a factor of *n*.
- We have reduced the dominant computational task at each time step from solving a full linear system to solving a sparse linear system. For general complex networks with no exploitable structure, it is difficult to be precise about the resulting gain, but we note that if a standard iterative scheme is used, then the cost of each matrix-vector multiplication is reduced by a factor of *n*.

### Comparing top *K* lists

The main goal of this work is to match the broadcast ranking of the nodes in an evolving network using a sparse approximation to the dynamic communicability matrix. As usual in network science, we are not interested in matching exactly the rankings of all nodes in the network, but rather to accurately capture the top *K*≪*n* most influential broadcasters. Although there is no perfect way to summarize and compare rankings, it is clear that generic correlation coefficients like Pearson’s correlation coefficient or Kendall’s tau have the major drawback in this context that they treat entire vectors, and hence all network nodes.

In order to compare the top *K* entries of two ranking vectors, an appropriate index is the *intersection similarity* (Fagin et al. 2003). This quantity is defined as follows: given two ranked lists *x* and *y*, consider the top *K* entries of each, which we denote *x*_*K*_ and *y*_*K*_, respectively. Then, the top *K* intersection similarity between *x* and *y* is defined as

5 $$ \text{isim}_{K}(x,y) = \frac{1}{K}\sum_{i=1}^{K}\frac{\left|x_{i}\Delta y_{i}\right|}{2i},  $$

where *Δ* is the symmetric difference operator between two sets and |*S*| denotes the cardinality of the set *S*. When the sequences contained in *x* and *y* are completely different, the intersection similarity between the two is maximum and equals 1. On the other hand, when isim_*K*_(*x*,*y*)=0 for all *K*, then the two lists are identical.

It happens sometimes that the two lists differ in the *order*, but not in the *set of labels* of the nodes appearing in them. Behaviour of this type can be easily spotted by looking at the quantity:

$$\ell_{K}(x,y) = K\cdot\text{isim}_{K}(x,y) - (K-1)\cdot\text{isim}_{K-1}(x,y), \quad K=2,3,\ldots $$

From (5) we have

$$\ell_{K}(x,y) = \frac{\left|x_{K}\Delta y_{K}\right|}{2K}, $$

and hence if *ℓ*_*K*_(*x*,*y*)=0 for some *K* we know that *x*_*K*_ and *y*_*K*_ are permutations of the same set of nodes.

### Relationship with the Jaccard index

The *Jaccard index* (Jaccard 1901) quantifies the similarity between two sample sets by measuring the percentage of elements that belong to both sets, thus ignoring the ordering of the elements. It is defined, for two sets *x* and *y*, as

$$J(x,y) = \frac{|x\cap y|}{|x \cup y|}\in [0,1] $$

and it equals 1 when the two sets coincide. It can be related to the intersection similarity and to the index *ℓ*_*k*_ through the *Jaccard distance*. This measure is defined for two sample sets as

$$d(x,y) = 1 - J(x,y) = \frac{|x\Delta y|}{|x \cup y|} $$

and hence

$$\ell_{K} \leq d(x_{K},y_{K}) = 1 - J(x_{K},y_{K}). $$

An easy computation thus shows that

$$\text{isim}_{K}(x,y) \leq 1 - \frac{1}{K}\sum_{i=1}^{K} J(x_{i},y_{i}). $$

## Numerical tests

Our tests were performed on three different datasets with various values of the parameter *α*. The dataset Enron is available at (Leskovec 2014) and contains daily information over 1138 days starting 11 May 1999 representing emails between 151 Enron employees, including to, cc, and bcc. Many of the directed adjacency matrices are empty, meaning that there are days during which no emails are sent. The largest spectral radius is *ρ* ^∗^=4.17, thus the upper limit for *α* is 0.24.

The undirected Real dataset is from (Eagle and Pentland 2006). Here, we have 106 nodes representing people interacting over 365 days. In each of the 365 days interaction occurs when two nodes communicate by telephone at least once. Here *ρ* ^∗^=8.22 and thus we have to impose *α*<0.12.

Finally, the dataset FBsoc (Opsahl 2009; Opsahl and Panzarasa 2009) represents a Facebook-like Social Network originating from an online community of students at the University of California. The directed dataset contains the 1899 users who sent or received at least one message over a period of 191 days starting 19 April 2004. The largest spectral radius is *ρ* ^∗^=7.59 and hence *α*<0.13.

In all tests, unless otherwise specified we allowed for a number of nonzeros proportional to $N = c\overline {n}$, where $\overline {n} = n + \frac {1}{M+1}\sum _{k=0}^{M}{\texttt {nnz}}\left (A^{[k]}\right)$ and *c*=10. This is motivated by our aim to work only with matrices whose sparsity level is compatible with that of the individual network time slices.

All experiments were performed using MATLAB Version 9.1.0.441655 (R2016b) on an HP EliteDesk running Scientific Linux 7.3 (Nitrogen), a 3.2 GHz Intel Core i7 processor, and 4 GB of RAM.

### Illustrative test with Enron dataset

Before testing the performance of (4), in this subsection we discuss the effect of including the multiplication by *m*_*k*_. In “Sparsification” section we argue that setting *m*_*k*_≡1 for all *k*=0,1,…,*M* in (4) may lead to poor results. Clearly, this is not always the case, but, as we will see here, this choice together with a compounding choice of the downweighting parameter *α*, may result in a complete misplacement of the top ranked broadcasters in the network.

**Fig. 1:**
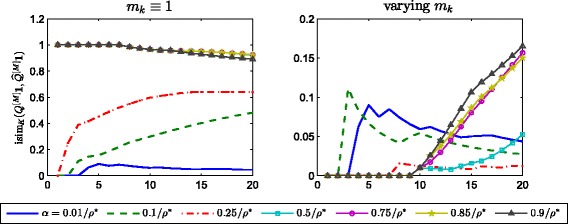
Evolution of isim_*K*_. Evolution of the intersection similarity $\text {isim}_{K}\left (Q^{[M]}\mathbf {1},\widehat {Q}^{[M]}\mathbf {1}\right)$ versus *K*, for different choices of the downweighting parameter *α*. *Left*: *m*_*k*_≡1. *Right*: *m*_*k*_ is set at each iteration as the smallest nonzero entry of $\left \lfloor \widehat {Q}^{[k-1]}\left (I+\alpha A^{[k]}\right) \right \rfloor _{\theta _{k}}$. Note the difference in vertical axis range

**Fig. 2:**
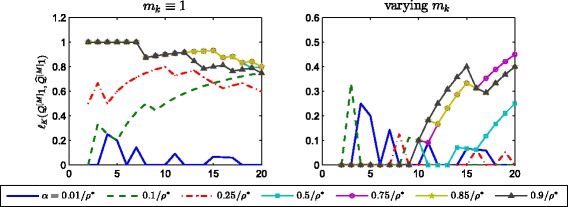
Evolution of *ℓ*_*K*_. Evolution of $\ell _{K}\left (Q^{[M]}\mathbf {1},\widehat {Q}^{[M]}\mathbf {1}\right)$ versus *K*, for different choices of the downweighting parameter *α*. *Left*: *m*_*k*_≡1. *Right*: *m*_*k*_ is set at each iteration as the smallest nonzero entry of $\left \lfloor \widehat {Q}^{[k-1]}\left (I+\alpha A^{[k]}\right) \right \rfloor _{\theta _{k}}$. Note the difference in vertical axis range

We compute the broadcast centrality vector *Q* ^[*M*]^ **1** and our approximation vector $\widehat {Q}^{[M]}\mathbf {1}$ for seven different values of the downweighting parameter:

$$\alpha = \frac{0.01}{\rho^{*}}, \frac{0.1}{\rho^{*}},\frac{0.25}{\rho^{*}},\frac{0.5}{\rho^{*}}, \frac{0.75}{\rho^{*}}, \frac{0.85}{\rho^{*}}, \frac{0.9}{\rho^{*}}, $$

where *ρ* ^∗^=4.17 is the largest spectral radius among the spectral radii of the matrices *A* ^[*k*]^, *k*=0,1,…,*M*. Figure 1 displays the evolution of the intersection similarity between the top *K*=1,2,…,20 entries of the vectors *Q* ^[*M*]^ **1** and $\widehat {Q}^{[M]}\mathbf {1}$ versus *K* for the different values of *α*. The left plot contains the results when *m*_*k*_≡1, while the right plot contains the results when *m*_*k*_ is adapted by setting it to be equal to the smallest nonzero entry of the matrix $\left \lfloor \widehat {Q}^{[k-1]}\left (I+\alpha A^{[k]}\right)\right \rfloor _{\theta _{k}}$ at each iteration. The results for the evolution of $\ell _{K}\left (Q^{[M]}\mathbf {1},\widehat {Q}^{[M]}\mathbf {1}\right)$ are displayed in Fig. 2.

These results show that when *m*_*k*_≡1 the intersection similarity and the value of *ℓ*_*K*_ between the two vectors can be maximum even when comparing only a few top ranked nodes for *α* as small as 0.5/*ρ* ^∗^. The right hand plots in the two figures show how an adaptive choice of *m*_*k*_ can work successfully over a wide range of *α* choices.

### Enron dataset

**Fig. 3:**
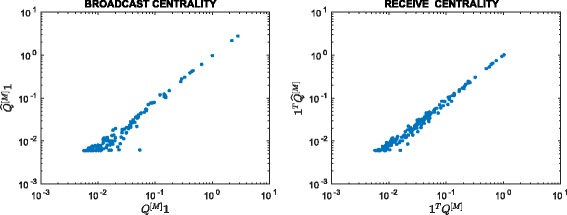
Scatter plot. Enron: approximation using (4) with *α*=0.01

**Fig. 4:**
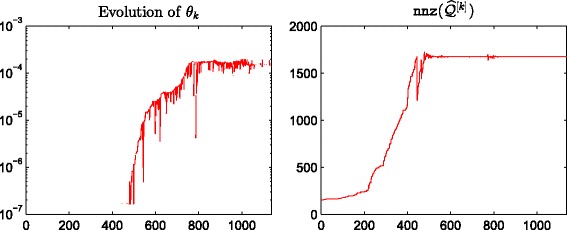
Evolution of *θ*_*k*_ and nnz(*Q* ^[*k*]^). Enron: behaviour of *θ*_*k*_ and the number of nonzerso in *Q* ^[*k*]^ with respect to *k*, using *α*=0.01

**Fig. 5:**
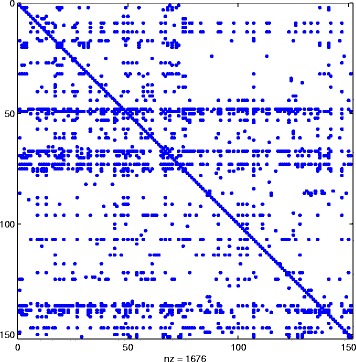
Sparsity pattern. Enron: sparsity pattern of the final matrix $\widehat {Q}^{[M]}$ computed using (4) with *α*=0.01

We now assess the effectiveness of iteration (4) at approximating the broadcast centrality rankings. For the Enron dataset we used *α*=0.01. The dynamic communicability matrix was computed in 2.62 s. The number of nonzero entries in this matrix is nnz(*Q* ^[*M*]^)=21097. Note that *n* ^2^=22801, so the matrix is 92.5*%* full. Figure 3 scatter plots the resulting approximation to the broadcast and receive centrality vectors against *Q* ^[*M*]^ **1** and **1** ^*T*^ *Q* ^[*M*]^, respectively. We observe a good linear correlation at the high end for both cases, indicating that our method correctly identifies important nodes. In Fig. 4 we plot the evolution of the thresholding parameters *θ*_*k*_ and of the sparsity of the approximation matrix $\widehat {Q}^{[k]}$ as *k* varies. We point out that our thresholding function sets to zero all entries of the matrix that are smaller than or equal to *θ*_*k*_, and this is the reason why we can find “drops” in the number of nonzeros in $\widehat {Q}^{[k]}$, even after the desired sparsity has been reached at around *k*=500. In Fig. 5 we display the sparsity pattern, i.e., the pattern of non-zeros, of the final approximating matrix $\widehat {Q}^{[M]}$, which was computed in 2.07 s.

Here, the number of nonzeros is =1676, so the level of sparsity has been reduced to around 7.4*%*. In Table 1 we list the top 10 ranked nodes according to the broadcast centrality. The first row contains the true result, obtained by ranking the nodes according to *Q* ^[*M*]^ **1**; in the second row we list the top 10 broadcasters according to the ranking derived from $\widehat {Q}^{[M]}\mathbf {1}$ and, finally, the last row displays the result obtained when the nodes are ranked according to their aggregate out-degree: $\sum _{k=0}^{M}A^{[k]}\mathbf {1}$. As *α*→0, the ranking obtained using the dynamic communicability matrix approaches that obtained using the aggregate out-degree; see, e.g., (Chen et al. 2016; Grindrod et al. 2011). Clearly, however, *α*=0.01 is not close enough to zero for this effect to be observed.

**Table 1:** Enron: Top 10 ranked nodes: exact, approximate and with aggregate out-degree

*Q* ^[*M*]^ **1**	48	67	147	73	13	50	137	49	9	139
$\widehat {Q}^{[M]}\mathbf {1}$	48	67	147	73	13	50	137	49	9	139
Out-degree	67	50	141	13	48	69	107	147	73	70

Tables 2 and 3 contain the values of $\text {isim}_{K}\left (Q^{[M]}\mathbf {1},\widehat {Q}^{[M]}\mathbf {1}\right)$ for *K*=1,2,…,20 and $\ell _{K}\left (Q^{[M]}\mathbf {1},\widehat {Q}^{[M]}\mathbf {1}\right)$ for *K*=2,3,…,20. The new method correctly orders the top 11 broadcasters in the network and correctly identifies the top 20.

**Table 2:** Enron: intersection similarity between the top *K*=1,2,…,20 ranked nodes in *Q* ^[*M*]^ **1** and $\widehat {Q}^{[M]}\mathbf {1}$

*K*	1	2	3	4	5	6	7	8	9	10
isim_*K*_	0	0	0	0	0	0	0	0	0	0
*K*	11	12	13	14	15	16	17	18	19	20
isim_*K*_	0	0.01	0.02	0.03	0.03	0.03	0.03	0.03	0.03	0.03

**Table 3:** Enron: evolution of $\ell _{K}\left (Q^{[M]}\mathbf {1},\widehat {Q}^{[M]}\mathbf {1}\right)$ for *K*=2,3,…,20

*K*	1	2	3	4	5	6	7	8	9	10
*ℓ*_*K*_	-	0	0	0	0	0	0	0	0	0
*K*	11	12	13	14	15	16	17	18	19	20
*ℓ*_*K*_	0	0.08	0.15	0.14	0.07	0	0.06	0	0.05	0

### Real dataset

**Fig. 6:**
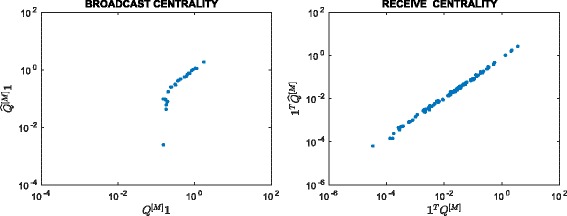
Scatter plot. Real: approximation using (4) with *α*=0.06

**Fig. 7:**
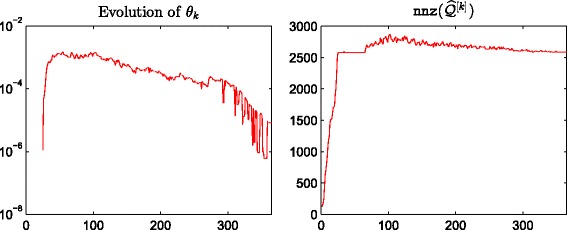
Evolution of *θ*_*k*_ and nnz(*Q* ^[*k*]^)Real: behaviour of *θ*_*k*_ and the number of nonzerso in *Q* ^[*k*]^ with respect to *K*, using *α*=0.06

**Fig. 8:**
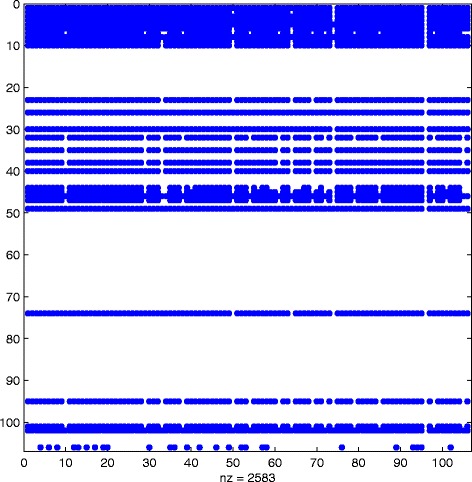
Sparsity pattern. Real: sparsity pattern of the final matrix $\widehat {Q}^{[M]}$ computed using (4) with *α*=0.06

We move on to the Real dataset. The value of the downweighting parameter used in this subsection is *α*=0.06<0.12=1/*ρ* ^∗^. The dynamic communicability matrix is completely full, as nnz(*Q* ^[*M*]^)=11236=*n* ^2^. We refer to Figs. 6, 7 and 8 for scatter plots of the ranking vectors and our approximation, the evolution of *θ*_*k*_ and ${\texttt {nnz}}\left (\widehat {Q}^{[k]}\right)$, and the sparsity pattern of $\widehat {Q}^{[M]}$. The original dynamic communicability matrix was computed in 0.34 s, while the final approximating matrix $\widehat {Q}^{[M]}$ was obtained in 0.71 s. We view the small size of the dataset as the main reason for the increase in computational time, which is now dominated by the selection of the parameters at each time step.

We see in Fig. 6 that the highly ranked nodes are well approximated. Even though the original dynamic communicability matrix is full, we see from the zero rows in Fig. 8 that many nodes have no activity recorded after our approximation method is applied. Overall, $\widehat {Q}^{[M]}$ has 2583 nonzeros, corresponding to 23% sparsity.

Table 4 lists the top 10 ranked nodes according to the broadcast centrality. As before, the first row contains the true result, obtained by ranking the nodes according to *Q* ^[*M*]^ **1**; in the second row we list the top 10 broadcasters according to the ranking derived from $\widehat {Q}^{[M]}\mathbf {1}$ and, finally, the last row displays the result obtained when the nodes are ranked according to their aggregate out-degree. As expected, the out-degree does not identify correctly the top broadcasters in the network. By contrast, the new approximation method correctly identifies the top 10 broadcasters.

**Table 4:** Real: Top 10 ranked nodes: exact, approximate and with aggregate out-degree

*Q* ^[*M*]^ **1**	5	102	8	26	49	46	3	4	1	30
$\widehat {Q}^{[M]}\mathbf {1}$	5	8	102	26	49	46	3	4	1	30
out-degree	5	8	4	2	3	20	40	6	23	53

Tables 5 and 6 contain the values of $\text {isim}_{K}\left (Q^{[M]}\mathbf {1},\widehat {Q}^{[M]}\mathbf {1}\right)$ for *K*=1,2,…,20 and $\ell _{K}\left (Q^{[M]}\mathbf {1},\widehat {Q}^{[M]}\mathbf {1}\right)$ for *K*=2,3,…,20. The results in Table 5 indicate that the new method performs well, in the sense that isim_*K*_ is small for all *K*. Further Table 6 shows that iteration (4) is able to correctly identify the top 19 broadcasters, albeit in the wrong order.

**Table 5:** Real: intersection similarity between the top *K*=1,2,…,20 ranked nodes in *Q* ^[*M*]^ **1** and $\widehat {Q}^{[M]}\mathbf {1}$

*K*	1	2	3	4	5	6	7	8	9	10
isim_*K*_	0	0.25	0.17	0.13	0.10	0.08	0.07	0.06	0.06	0.05
*K*	11	12	13	14	15	16	17	18	19	20
isim_*K*_	0.05	0.04	0.04	0.04	0.04	0.04	0.03	0.03	0.03	0.03

**Table 6:** Real: evolution of $\ell _{K}\left (Q^{[M]}\mathbf {1},\widehat {Q}^{[M]}\mathbf {1}\right)$ for *K*=2,3,…,20

*K*	1	2	3	4	5	6	7	8	9	10
*ℓ*_*K*_	-	0.50	0	0	0	0	0	0	0	0
*K*	11	12	13	14	15	16	17	18	19	20
*ℓ*_*K*_	0	0	0	0.07	0	0	0	0	0	0.05

**Fig. 9:**
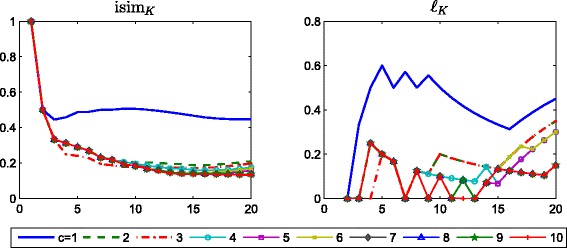
Vary *c*. Real: behaviour of isim_*K*_ and *ℓ*_*K*_ with respect to *K*, using *α*=0.1

Increasing *α* to =0.1, we look at the effect of varying *c*, the factor used to determine the sparsity of the final matrix. Figure 9 shows the evolution of $\text {isim}_{K}\left (Q^{[M]}\mathbf {1},\widehat {Q}^{[M]}\mathbf {1}\right)$ and $\ell _{K}\left (Q^{[M]}\mathbf {1},\widehat {Q}^{[M]}\mathbf {1}\right)$ versus *K* when *c*=1,2,…,10. Larger values of *c* were tested (*c*=15,17) and found to provide the same results as *c*=7,8,9. We conclude that for this data set the results are not sensitive to the choice of *c*, and that *c*=10 is a reasonable level.

### FBsoc dataset

**Fig. 10:**
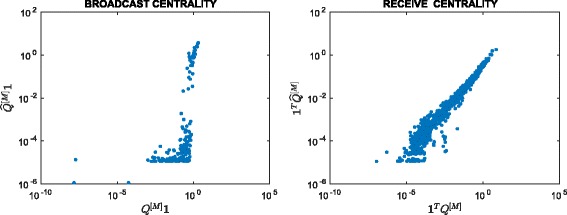
Scatter plot. FBsoc: approximation using (4) with *α*=0.1

**Fig. 11:**
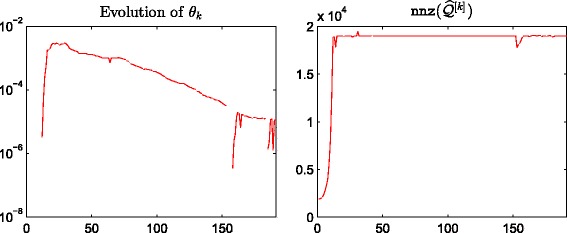
Evolution of *θ*_*k*_ and nnz(*Q* ^[*k*]^)FBsoc: behaviour of *θ*_*k*_ and the number of nonzerso in *Q* ^[*k*]^ with respect to *K*, using *α*=0.1

**Fig. 12:**
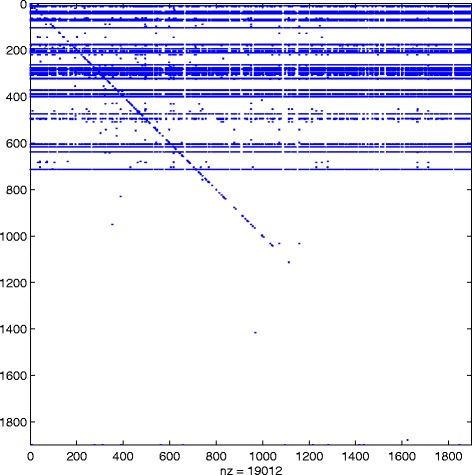
Sparsity pattern. FBsoc: sparsity pattern of the final matrix $\widehat {Q}^{[M]}$ computed using (4) with *α*=0.1

The last test has been performed on the dataset FBsoc with *α*=0.1. Here *Q* ^[*M*]^ has 1872718 nonzeros, with *n* ^2^=3606201, corresponding to 52% nonzeros. We refer to Figs. 10, 11 and 12 for the plots of centralities, *θ*_*k*_ and ${\texttt {nnz}}\left (\widehat {Q}^{[k]}\right)$ evolution and the sparsity pattern of $\widehat {Q}^{[M]}$. The number of nonzeros in the final approximation $\widehat {Q}^{[M]}$ is 19012, giving 0.53*%* sparsity. This matrix was computed in 339.72 s, while the computation of *Q* ^[*M*]^ required 398.39 s.

We note in Fig. 10 that, at least visually, the ranking of highly central nodes seems less successful than for the previous two data sets.

Table 7 lists the top 10 ranked nodes according to the broadcast centrality (first row), our approximation (second row), and the aggregate out-degree (third row). Using the aggregate out-degree returns poor results. Our method exactly matches the top two broadcasters and is able to identify a few other top nodes, even if in the wrong order. This observation is confirmed by the values of $\text {isim}_{K}\left (Q^{[M]}\mathbf {1},\widehat {Q}^{[M]}\mathbf {1}\right)$ and $\ell _{K}\left (Q^{[M]}\mathbf {1},\widehat {Q}^{[M]}\mathbf {1}\right)$, which are generally low (see Tables 8 and 9). Indeed, our method correctly matches only the two top broadcasters in the network. The values of *ℓ*_*K*_ are small for all *K*, meaning that we are mis-ordering nodes but still identifying most of the influential ones. For example, the value of *ℓ*_20_=0.20 shows that we are correctly identifying 16 out of the 20 top broadcasters in the network.

**Table 7:** FBsoc: Top 10 ranked nodes: exact, approximate and with aggregate out-degree

*Q* ^[*M*]^ **1**	9	103	212	41	263	321	400	372	281	36
$\widehat {Q}^{[M]}\mathbf {1}$	9	103	41	212	400	321	36	372	44	713
out-degree	32	598	372	1624	42	103	713	638	495	617

**Table 8:** FBsoc: intersection similarity between the top *K*=1,2,…,20 ranked nodes in *Q* ^[*M*]^ **1** and $\widehat {Q}^{[M]}\mathbf {1}$

*K*	1	2	3	4	5	6	7	8	9	10
isim_*K*_	0	0	0.11	0.08	0.11	0.12	0.12	0.12	0.13	0.14
*K*	11	12	13	14	15	16	17	18	19	20
isim_*K*_	0.14	0.14	0.15	0.15	0.15	0.16	0.16	0.17	0.17	0.17

**Table 9:** FBsoc: evolution of $\ell _{K}\left (Q^{[M]}\mathbf {1},\widehat {Q}^{[M]}\mathbf {1}\right)$ for *K*=2,3,…,20

*K*	1	2	3	4	5	6	7	8	9	10
*ℓ*_*K*_	-	0	0.33	0	0.20	0.17	0.14	0.13	0.22	0.20
*K*	11	12	13	14	15	16	17	18	19	20
*ℓ*_*K*_	0.09	0.17	0.23	0.22	0.20	0.19	0.24	0.28	0.26	0.20

**Fig. 13:**
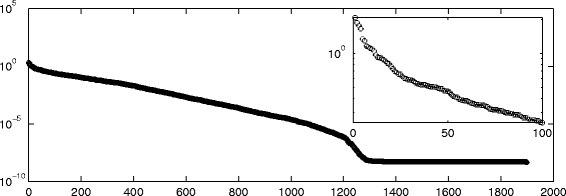
Ordered broadcast vector. FBsoc: ordered broadcast vector *Q* ^[*M*]^ **1** (*α*=0.1). Inset: broadcast centralities of the top 100 nodes in the network

The results for this network are not as good as those obtained for the previous two treated in this paper. To investigate further, in Fig. 13 we plot, with a logarithmic vertical axis, the broadcast centralities of nodes in non-increasing order (*α*=0.1), while the inset contains the broadcast centralities of the top 100 nodes in the network, again in non-increasing order. This plot clearly shows that beyond the top few nodes (say, 4, 5 or 6), the broadcast centralities are tightly packed. Indeed, if we consider the broadcast centrality vector components in non-increasing order and compute the average difference between two subsequent entries, we obtain 0.001 for this dataset, as opposed to 0.018 for Enron and 0.016 for the Real dataset. Moreover, the largest gap for FBsoc is 0.31, while it is 1.20 for Enron and 0.61 for Real. We view this clustering of the centrality values as the main reason for the degradation in performance on this dataset.

## Further reduction

In the previous Sections we have derived a sparsification technique that delivers accurate approximations to the full-matrix broadcast centrality rankings. The iteration described in (4) requires the selection, at each time stamp, of two parameters: *θ*_*k*_ and *m*_*k*_. In this Section we discuss three different ways of selecting these parameters and provide the results of some numerical tests in order to assess the effectiveness of the proposed variations of (4). From (4) it is clear that *m*_*k*_>*θ*_*k*_. In order to reduce the number of parameters we can thus select at each time stamp *m*_*k*_=*θ*_*k*_, which is smaller than the smallest postive entry of $\widehat {\mathcal {Q}}^{[k]}:=\left \lfloor \widehat {Q}^{[k-1]}\left (I+\alpha A^{[k]}\right)\right \rfloor _{\theta _{k}}$. Unless the smallest nonzero entry of $\widehat {\mathcal {Q}}^{[k]}$ is much larger than *θ*_*k*_, this selection of *m*_*k*_ should provide results as accurate as the one we obtained using (4). The new iteration is thus:

6a $$ \widehat{Q}^{[k]} = \left\lfloor \widehat{Q}^{[k-1]}\left(I+\alpha A^{[k]}\right)\right\rfloor_{\theta_{k}} + \theta_{k}{\mathcal{A}}^{[k]}, \quad k=0,1,\ldots,M,  $$

followed by normalization, where $\widehat {Q}^{[-1]}= I$ and ${\mathcal {A}}^{[k]} = \alpha W^{[k]}A^{[k]}$ as before.

In order to further reduce the number of parameters, one can use a fixed value for the thresholding parameter *θ*_*k*_. Indeed, one may want to retain only the information contained in the iteration matrix that is above a certain value, or might have additional information about the structure of the matrix which allows one to customize the choice of the thresholding parameter. In these cases, one can use a fixed value for *θ*_*k*_≡*θ*:

6b $$ \widehat{Q}^{[k]} = \left\lfloor \widehat{Q}^{[k-1]}\left(I+\alpha A^{[k]}\right)\right\rfloor_{\theta} + m_{k}{\mathcal{A}}^{[k]}, \quad k=0,1,\ldots,M,  $$

followed by normalization, where $\widehat {Q}^{[-1]}= I$ and ${\mathcal {A}}^{[k]} = \alpha W^{[k]}A^{[k]}$ as before. It is easy to see that the parameter *θ* cannot exceed the value of *α*. Indeed, if this was the case and *θ*≥*α*, then $\widehat {Q}^{[k]} = I$ for all *k*=0,1,…,*M*, since $\widehat {Q}^{[0]} = I$ and thus $\left \lfloor \widehat {Q}^{[k-1]}\left (I+\alpha A^{[k]}\right)\right \rfloor _{\theta } = I$ and *W* ^[*k*]^=0 for all *k*=1,2,…*M*. Therefore, *θ*<*α*.

Finally, the two approaches previously described in (6a) and (6b) can be combined to obtain:

6c $$ \widehat{Q}^{[k]} = \left\lfloor \widehat{Q}^{[k-1]}\left(I+\alpha A^{[k]}\right)\right\rfloor_{\theta} + \theta{\mathcal{A}}^{[k]}, \quad k=0,1,\ldots,M,  $$

returning an iteration that only requires the selection of one, fixed, thresholding parameter *θ*<*α*.

We tested the performance of the methods just described on the datasets Enron and Real. Before moving on to the discussion of the ranking performance of these variants of (4), we want to list the timings required for their computation. Concerning the first dataset, the timings required for the computation of the approximating matrices are 1.98 s for (6a), 1.97 s for (6b) and 1.83 s for (6c). Concerning the Real dataset, the computations were carried out in 0.67 s for (6a), 0.32 s for (6b) and 0.28 s for (6c). As one would expect, the time required by the methods decreases with the number (and type) of parameters that need to be estimated at each iteration.

**Fig. 14:**
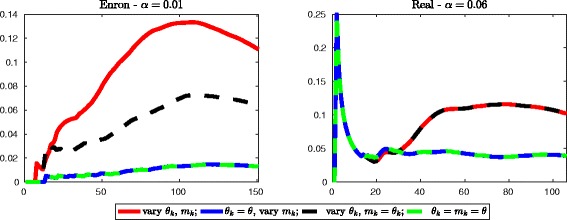
Further reduction. Evolution of the intersection similarity between $\widehat {Q}^{[M]}\mathbf {1}$ and *Q* ^[*M*]^ **1** when the former is computed using one among (4), (6a), (6b), or (6c). In the computations, *θ*=10^−5^ for the network Enron and *θ*=10^−4^ for the network Real

Figure 14 displays the intersection similarity between the top *K*=1,2,…,*n* entries of the vectors *Q* ^[*M*]^ **1** and $\widehat {Q}^{[M]}\mathbf {1}$, when this latter is computed using the iterations previously described (4), (6a), (6b), and (6c). The results are displayed for the networks Enron (*α*=0.01) and Real (*α*=0.06). When iteration (6b) or (6c) is used, we set *θ*=10^−5^ for the network Enron and *θ*=10^−4^ for the network Real. These values of *θ* correspond to the order of magnitude of the average value of the thresholding parameters used in “Numerical tests” section. The methods are seen to perform well. In both cases we obtain an intersection similarity that is, at each step, lower than 0.25. The new iterations return results that are, in the worst case scenario, as good as those obtained using (4). The number of nonzeros in the resulting matrix $\widehat {Q}^{[M]}$ for the dataset Enron is 5847 when (6b) or (6c) is used, and 1676 for (6a). In the first two cases we are achieving better results than those previously obtained, but the level of sparisty of $\widehat {Q}^{[M]}$ is 25.6*%*; therefore, a better approximation of the broadcast centrality vector is obtained because we are retaining more nonzeros in the matrix used to perform the computation.

Overall, the method described in (6a) seems to be the one performing best, since it returns a matrix that has the same level of sparsity of the one obtained using (4), but the resulting ranking vector better matches *Q* ^[*M*]^ **1**.

If we now look at the results obtained for the network Real, we observe again that the best performance, in terms of the intersection similarity between the top *K* entries of $\widehat {Q}^{[M]}\mathbf {1}$ and *Q* ^[*M*]^ **1**, is achieved using iterations (6b) and (6c); the iteration described in (6a) performs better than the original one. If we now look at the level of sparsity of the final approximation matrices, we have that overall $\widehat {Q}^{[M]}$ computed using (6a) has 2581 nonzeros, corresponding to 23% sparsity. Both (6b) and (6c) return matrices that have 4243 nonzeros, corresponding to 40.4*%* sparsity. Thus, as before, these two latter methods return better approximation to the ranking vector because they are retaining more information in the matrices used in the computations.

Overall, a good compromise seems to be the use of (6a), which returns comparable results to those obtained by the original iteration while retaining the same level of sparsity.

## Conclusions

Time-dependency adds an extra dimension to network science computations, potentially causing a dramatic increase in both strorage requirements and computation time. In the case of Katz-style centrality measures, which are based on the solution of linear algebraic systems, allowing for the arrow of time leads naturally to full matrices that keep track of all possible routes for the flow of information. Such a build-up of intermediate data can make large-scale computations infeasible. In this work, we derived a sparsification technique that delivers accurate approximations to the full-matrix centrality rankings, while retaining the level of sparsity present in the network time-slices. With the new algorithm, as we move forward in time the storage cost remains fixed and the computational cost scales linearly, so the overall task is equivalent to solving a single Katz-style problem at each new time point. We also proposed three variants of this algorithm that require the computation of a smaller number of parameters. In particular, one of these variants requires only one parameter and returns rankings that are comparable with those provided by the original algorithm.
